# Cooling-Triggered Release of Celecoxib from Implantable Alginate-Soluplus Composite Devices

**DOI:** 10.1021/acsbiomaterials.5c00867

**Published:** 2025-08-25

**Authors:** Romario Lobban, Michael Carroll, Victoria Vest, Josh T. McCune, Sarah Hall, Fang Yu, Md. Jashim Uddin, Lawrence J. Marnett, Craig L. Duvall, Leon M. Bellan

**Affiliations:** † Department of Mechanical Engineering, 5718 Vanderbilt University, 2201 West End Avenue, Nashville, Tennessee 37235, United States; ‡ Division of General Engineering, Vanderbilt University, 2201 West End Avenue, Nashville, Tennessee 37235, United States; § Department of Biomedical Engineering, Vanderbilt University, 2201 West End Avenue, Nashville, Tennessee 37235, United States; ∥ Department of Biochemistry, Vanderbilt University School of Medicine, 1161 21st Avenue South, Nashville, Tennessee 37232, United States; ⊥ Department of Chemistry, Vanderbilt University School of Medicine, 1161 21st Avenue South, Nashville, Tennessee 37232, United States; # Department of Pharmacology, Vanderbilt University School of Medicine, 1161 21st Avenue South, Nashville, Tennessee 37232, United States; 7 Department of Chemical and Biomolecular Engineering, Vanderbilt University, 2201 West End Avenue, Nashville, Tennessee 37235, United States; 8 Department of Ophthalmology and Visual Sciences, Vanderbilt University, 2201 West End Avenue, Nashville, Tennessee 37235, United States

**Keywords:** nonsteroidal anti-inflammatory drugs (NSAIDs), pain-relief, postoperative pain-relief, stimulus-triggered drug delivery, cooling-triggered drug delivery, Soluplus, alginate

## Abstract

Currently, on-demand treatment of pain (both chronic and acute) is primarily achieved using opioids that are delivered systemically. Unfortunately, these drugs are highly addictive; over 5 people per hour die from opioid abuse in the US alone. A safer, nonsystemic mechanism for pain relief is therefore needed. Nonsteroidal anti-inflammatory drugs (NSAIDs) have been explored for this purpose; they are nonaddictive, provide excellent pain relief, and can be delivered locally to minimize dosage and systemic side effects. However, an on-demand release method is needed to make local delivery of these drugs a viable, convenient replacement for opioids; external stimulus-triggered release from an implantable depot is one approach. Stimuli such as heat, light, ultrasound, and RF electromagnetic radiation have been used to trigger release of various drugs from implantable drug depots; however, these require energy input and complex apparatus and are thus not comparable to the ease of oral administration. We propose localized cooling as a safe, convenient stimulus. As icepacks are already widely applied to temporarily ease local pain, introducing a drug delivery mechanism switched “ON” by cooling could enable long duration, enhanced pain relief triggered by a method with which patients are already familiar. Herein, we demonstrate that cooling-triggered release of NSAIDs can be achieved by leveraging the gel-to-sol transition exhibited by physically cross-linked thermoresponsive polymer hydrogels upon cooling below their lower critical solution temperature (LCST). We demonstrate and characterize cooling-triggered release in simulated body fluid, in cell culture, in explanted tissue, and in a live animal wound model. We show that hydrogels loaded with an NSAID (Celecoxib) can be combined with a nonthermoresponsive membrane material to create implantable devices that demonstrate up to a ∼40× increase in drug release rate upon mild cooling (29 °C) and that support multiple cycles of triggered release. These results demonstrate that cooling-triggered release of therapeutics is a promising concept that could allow patients to use a familiar method (applying an icepack to pain points) to achieve enhanced pain relief.

## Graphical abstract

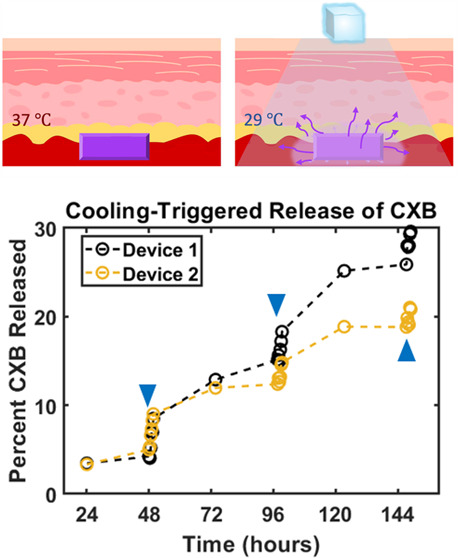

## Introduction

Nonsteroidal anti-inflammatory drugs (NSAIDs) are commonly used to relieve pain and inflammation associated with conditions such as headaches, postoperative recovery, and musculoskeletal disorders like gout and rheumatoid arthritis, among others.^1,2^ These analgesic and anti-inflammatory properties are primarily attributed to the ability of NSAIDs to inhibit cyclooxygenase (COX) activity.^1–3^ Among the most well-known examples of NSAIDs are Ibuprofen, Aceclofenac, Celecoxib, Diclofenac, Naproxen and Indomethacin.^1–3^ These agents have traditionally been administered orally; however, recent research has generated increasing interest in the development and application of alternative, more localized methods of drug delivery. Such approaches include transcutaneous, subcutaneous, and intra-articular delivery routes, which offer the potential for targeted therapeutic effects, reduced systemic side effects, and improved patient outcomes compared to conventional oral administration.^4,5^ Such approaches promise to reduce side effects associated with systemic delivery (such as gastrointestinal irritation) and minimize drug–drug interactions.^2,4^

Celecoxib (CXB) is an NSAID known for its selective inhibition of cyclooxygenase-2 (COX-2), an enzyme involved in the inflammatory process. Due to its specific mechanism of action and reduced gastrointestinal side effects compared to nonselective NSAIDs, celecoxib has been investigated as a promising candidate for local drug delivery systems. Such approaches aim to enhance therapeutic efficacy at the target site while minimizing systemic exposure and associated adverse effects, thereby improving patient outcomes in conditions characterized by localized inflammation or pain.^6–8^ After its introduction in 1998, it became one of the most prescribed drugs for treating acute pain associated with osteoarthritis, rheumatoid arthritis, and ankylosing spondylitis;^9,10^ its ability to selectively inhibit COX-2 over COX-1 allows for lower levels of gastrointestinal irritation without reducing its effectiveness.^8,11^ However, cardiovascular risks observed in similar drugs (COX-2 selective NSAIDs) and CXB itself remain a major concern.^1,12^ Recent studies suggest local delivery of CXB could allow for smaller doses and lessened side effects. Tellegen et al.^13^ described local delivery of CXB to joints via an injectable, macroscopic PCLA–PEG–PCLA hydrogel for reduction of osteoarthritis (OA)-induced inflammation; they observed reduced inflammation with an absence of both systemic and local adverse effects. Similarly, Janssen et al.^14^ also found local delivery of CXB to arthritic joints to be effective in reducing inflammation without adverse effects.

CXB has also been shown to reduce pain and inflammation more broadly.^8,15–17^ Qin et al.^16^ used nanoparticles injected intramuscularly (into rats) for extended release of CXB and thereby demonstrated analgesic effects lasting as long as 5 days. Zhao et al.^17^ showed that intraperitoneal injection of CXB significantly reduced both long and short-term formalin-induced pain and inflammation in a rat model. Such studies demonstrate that CXB has potential for treating pain and inflammation outside the realm of arthritis and other musculoskeletal disorders and that it may also reduce postoperative pain and inflammation^16^ and aid in recovery from dental procedures.^18^

Many approaches employed for local delivery of NSAIDs leverage passive, extended-release formulations to achieve high local drug concentrations over extended timeframes.^14,19,20^ However, to match the on-demand availability provided by oral delivery of NSAIDs, a method allowing patients to trigger release is necessary. Some prior work has demonstrated external stimulus-triggered release of NSAIDs from implanted devices. For example, Bariana et al.^21^ demonstrated radiofrequency-triggered release of Indomethacin from nanotube implants. Meanwhile, Gambles et al.^22^ demonstrated UV-triggered release of CXB. Such approaches show great promise for easy, on-demand release of NSAIDs; however, they generally require energy input and may not be easily applied at home due to the need for complex apparatus. Cooling, however, is a simple, easily applied trigger that is already widely applied for partial relief of pain and inflammation;^23,24^ it is therefore quite promising as a trigger for NSAID release. Cooling-triggered drug release has been explored by researchers to deliver anticancer^25,26^ or pain relief therapeutics^27^ from nano/micro scale reservoirs at temperatures ranging from 4 to 25 °C. Other previous work has demonstrated cooling-triggered release (at 22 °C) of the uncharged macromolecule Dextran from templated PNIPAM (poly n-isopropylacrylamide) microgels;^28^ the geometry of these microgels is such that cooling-induced swelling of the PNIPAM allows escape of the Dextran payload. Additionally, *in vivo* cooling-triggered release (at 25 °C) of cancer-targeting cytotoxins from NIPAM (n-isopropylacrylamide)-based nanoparticle complexes has also been demonstrated;^29^ upon cooling, the NIPAM-based nanoparticle loses (due to increased hydration and a drop in hydrophobicity) its engineered affinity for the cytotoxic peptide, Melittin, thereby allowing its release. However, to our knowledge, no studies have demonstrated cooling-triggered release of pain relief therapeutics from a macroscopic implant at temperatures ∼30 °C (closer to body temperature). Importantly, prior research clearly demonstrates that safe episodes of skin cooling (via application of ice) result in intramuscular temperatures only slightly below 30 °C;^23,24^ triggering at less extreme temperatures (∼30 °C) therefore extends the range of depths at which a reservoir can be placed. We also propose that use of a macroscopic implant is desirable when pain and inflammation are easily localized, such as for postoperative pain and inflammation; Xaracoll^30^ is an example of an FDA-approved, macroscopic implant for postoperative pain relief, though it is loaded with a local anesthetic, rather than an NSAID, and only provides passive, not triggerable, release.

To achieve cooling-triggered release of NSAIDs (as shown in Figure 1), we designed a formulation of NSAID-loaded Soluplus (thermoresponsive component) that is itself immobilized within an alginate matrix. Soluplus is a polyvinyl caprolactam copolymer (polyvinyl caprolactam/polyvinyl acetate/polyethylene glycol copolymer) produced by BASF. When in solution above its critical micellar concentration (CMC), it forms micelles that can be used to enhance solubility and bioavailability of highly hydrophobic drugs (like the NSAIDs CXB and Indomethacin etc. and other anti-inflammatory drugs like Curcumin).^31–35^ Soluplus is also known to exhibit lower critical solution temperature (LCST) behavior;^36,37^ when below its LCST, it exists as a solution, but when above its LCST it exhibits gel-like properties. This behavior results from micelles clumping together, rather than the formation of a more typical hydrogel network.^37^ We chose alginate as the matrix material because of its well-known biocompatibility and the mildness of the inflammatory response triggered by its implantation.^38,39^ Additionally, recent work^40^ has demonstrated modifications that further improve the biocompatibility of alginate. Furthermore, the degradability of alginate and alginate gels can be engineered via a variety of approaches.^41,42^ Our use of high-G (more guluronic acid units than mannuronic acid units; in our case >60% guluronic acid) alginate combined with 0.1% linear polyethylenimine (LPEI) ensured that the resulting alginate matrix maintained structural integrity despite prolonged exposure (at least 2 weeks) to the ions present in simulated body fluid. Importantly, this material did show signs of initial gel degradation at 2 weeks, likely via diffusion of calcium ions out of the gel by a mechanism previously described.^43,44^ Previous work has reported on this use of small amounts of LPEI^45^ to solve the issue of alginate erosion under physiological conditions. Such amounts of LPEI were shown to have only a very mild effect on biocompatibility while having a marked effect on gel longevity and robustness.^45^

**Figure 1:**
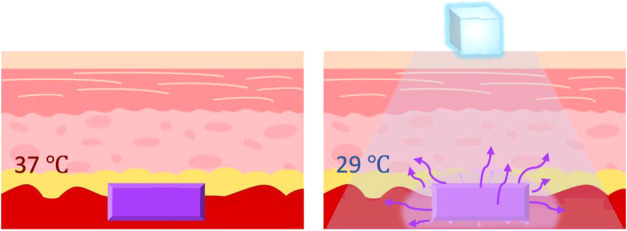
Cooling-triggered drug release from macroscopic implantable hydrogel composite device.

## Experimental Section

### Materials

Soluplus (*K*-value = 36) was provided to us as a gift from BASF. Pronova UP MVG sodium alginate (250 mPa) was purchased from Millipore Sigma. Celecoxib secondary standard (PHR-1683), Curcumin (C-1386), Nile red, DMSO­(BP231-1), and Linear poly­(ethylenimine) (LPEI; Polysciences PEI 25K), were all purchased from Millipore Sigma. Fluorocoxib A (FA; a fluorescently tagged NSAID) was synthesized as described by Uddin et al.^46^ In this work, FA is used to visualize cooling-triggered release and both *in vitro* and *in vivo* uptake of an NSAID payload released from composite devices. The chemical structures of compounds used in this manuscript are shown in Figure S1.

### Soluplus Solution Preparation

We mixed dry Soluplus with Nile red or Curcumin, then added the mixture to DI water and magnetically stirred for 3 min. For CXB-laden formulations, we mixed dry Soluplus and CXB; this solid mixture was then heated at 130 °C for 30 min, resulting in a fused, soft slab. This slab, upon cooling, hardened and was ground into fine pieces using a mortar and pestle and/or hammer (similar to the approach used by Homayouni et al.^31^). These pieces were then added to DI water and magnetically stirred for 3 min. Thermogravimetric Analysis (TGA) was used to confirm thermal stability of the CXB, Soluplus, and combination thereof under the conditions described above (Supplementary Data).

All magnetically stirred formulations (blanks, Nile red, Curcumin, and CXB-laden) were then homogenized using a Thinky mixer (Thinky ARE-250) (2 × 3 min cycles at 1800 rpm). These homogenized formulations were stored at 37 °C for 48 h, then homogenized once more (1 × 3 min cycle at 1800 rpm). Following the second homogenization, the mixtures were stored at 70 °C for 48 h. Upon removal from the 70 °C environment and re-equilibration to 37 °C for at least 6 h, the mixtures were observed to be clear/lightly translucent below their LCST and opaque above it; becoming clear upon cooling was used to confirm complete dissolution of the Soluplus and payload.^36,47^ The contents of the various formulations produced are shown in Table 1 of the results section. We used 30 mL of DI water for preparation of each formulation. Additionally, we used 16.2 g of Soluplus for all formulations. For loaded formulations, 0.065 g of Nile red, 0.81 g of Curcumin, or 1.296 g of CXB was used.

**Table 1:** Concentrations and Gel and Solution Points of Formulations Prepared^a^

formulation	water w/w%	Soluplus w/w%	payload w/w%|*w*_drug_/*w*_Soluplus_%	GP; SP (±1 °C)
blank Soluplus	65	35	0	36; 32
Nile red-laden Soluplus	65	35	0.14% Nile red|0.4%	35; 32
Curcumin-laden Soluplus	64	34	1.72% Curcumin|5%	32; 28
CXB-laden Soluplus	63	34	2.73% CXB|8%	35; 29^a^

a Values shown for Sol-CXB are taken from experiments using 1.25 rad/s oscillation frequency; the others were taken using 12.5 rad/s.

### Alginate-LPEI Preparation

Alginate solution was prepared in DI water and combined with LPEI solution to create a final formulation of 2.6 w/v% alginate and 0.1 w/v% LPEI. This solution was adjusted to a pH of ∼8 to prevent premature alginate cross-linking.

Specifically, we prepared 25 mL of 3.2% sodium alginate solution in DI water by stirring (using a magnetic stir bar) for an hour at 50 °C. Simultaneously, we prepared 5 mL of 0.6% LPEI solution in 4 mL of DI water + 1 mL of 0.1 M HCl (to aid LPEI dissolution), also using a magnetic stir bar. When both alginate and LPEI were fully dissolved, we slowly combined their solutions to make 30 mL of 2.67% alginate, 0.1% LPEI solution. During this combination, turbidity and alginate cross-linking were observed (due to the presence of LPEI and the low pH of the LPEI solution^48^). We then titrated 1 M NaOH into the mixture under continuous stirring at 50 °C until turbidity was no longer observed; basic conditions have previously been shown to minimize interaction between PEI and alginate.^48^ Next, we used a pH meter (Mettler Toledo SevenExcellence Multi Parameter) to measure the resulting pH. Often, we subsequently needed to add a small amount of 1 M HCl (∼0.1 mL) to the solution to achieve a pH of ∼8 at 50 °C (measured by pH meter). In total, formulations required less than 0.8 mL of NaOH and HCl solution; final alginate concentration therefore remained 2.6% (to the nearest 0.1%).

### Alginate/Soluplus Composite Fabrication

The alginate-LPEI solution was combined with a Soluplus solution in a 1.35:1 ratio. This mixture was stirred for 5 min at 70 °C, then poured into a mold (Figure 3A,B) with volume 0.38 mL. The mold was then submerged in previously warmed (70 °C) calcium chloride solution (60 mL; 3 w/v%) and the alginate allowed to cross-link for 30 min. The cross-linked composite device was then placed in 10 mL of simulated body fluid (SBF, prepared as described by Kokubo et al.^49^) at 35–37 °C (inside a Binder KB-115 incubator) and pH of ∼7.4 for 24 h (to allow for initial burst release). Briefly, SBF was previously prepared by dissolving NaCl (8 mg/mL), NaHCO_3_ (0.35 mg/mL), KCl (0.22 mg/mL), K_2_HPO_4_·3H_2_O (0.23 mg/mL), MgCl_2_·6H_2_O (0.31 mg/mL), CaCl_2_ (0.28 mg/mL), Na_2_SO_4_ (0.07 mg/mL), and (CH_2_OH)_3_CNH_2_ (6.1 mg/mL) into 1L of DI water including 44.5 mL 1 M HCl (to achieve a pH of ∼ 7.4; measured by pH-meter at 37 °C).

### Rheometry

Rheometry was used to find the LCST (both the gel and solution points) of all formulations (Table 1). All experiments were done in triplicate and performed on an Ares G2 rheometer with 20 mm parallel plate geometry and a gap of 1 mm. Parameters used to study each formulation were determined independently using preliminary amplitude and frequency sweeps at 35–37 and 23 °C (to capture behavior above and below the LCST, respectively); parameters were then chosen to lie within the linear viscoelastic region of a given formulation. Temperature ramps were performed on all formulations from 20 to 42 °C and back down to 26 °C with a ramp rate of 2 °C/min in both directions. Formulations were tested at a constant stress amplitude of 5 Pa and oscillation frequency of 12.5 rad/s, except CXB-laden formulations, which were tested at both 12.5 and 1.25 rad/s. As is typical, we defined the point at which storage modulus surpassed loss modulus during the upward temperature ramp as the gel point (GP) and the opposite as the solution point (SP).

### SEM

Composite devices were washed at 35–37 °C for 24 h then kept below their solution points for 1 h to allow some Soluplus to escape from the alginate matrix, thereby creating the “partially vacated” composite devices shown in Figure 3. Subsequently, the partially vacated device was plunged into liquid nitrogen and lyophilized for at least 24 h. This lyophilized sample was then coated with gold and observed using SEM (Zeiss Merlin). As controls, unvacated composites (not exposed to temperatures below the relevant solution point) and devices made with only alginate were subjected to the same procedure.

### Confocal Imaging

Composite devices (Nile red-laden) were fabricated and then immediately observed using a confocal microscope (Zeiss LSM 710).

### Dynamic Light Scattering (DLS)

Composite devices were allowed to release CXB-laden Soluplus into 10 mL SBF wells. Two mL portions of samples from these wells were placed into cuvettes and a Malvern Panalytical Zetasizer Ultra was used for dynamic light scattering (DLS) characterization of the released Soluplus micelles both above (allowing 10 min to equilibrate at 37 °C) the relevant GP and below (23 °C) the relevant SP.

### TEM

Composite devices were allowed to release pure or CXB-laden Soluplus into 10 mL SBF wells. Carbon coated 400 mesh TEM grids (01822-F, Ted Pella, Inc.) were treated with glow discharge for 1 min at 25 mA to increase hydrophilicity. To prep the grids, 5 μL of sample drawn from composite device release wells was added to carbon films and incubated for 1 min. The remaining sample was removed, then 5 μL of uranyl acetate (3 wt %) was added to negatively stain the particles. The negative stain was incubated for 1 min, then removed. The films were dried overnight at room temperature. The samples were imaged on a Tecnai Osiris TEM/STEM operating at 200 kV.

### Solubilization of Payloads Released from Composite Devices

Composite devices were allowed to release payload-laden Soluplus into 10 mL SBF wells at 29 °C. The total quantity of Nile red, Curcumin, or CXB released was then assessed using the methods described below. The wells were then allowed to equilibrate at room temperature for 24 h (with shaking). They were then centrifuged at 5000 rpm for 12 min (using a Sorvall Legend X1). One mL was then drawn from their top portions and filtered using a vacuum filter (Steriflip 0.22 μm). The filtered portion was then assessed to determine the extent of payload solubilization.

### *In Vitro* Cooling-Triggered Release

Composite devices were placed in 10 mL wells of SBF and gently shaken using an orbital rocker (Scilogex SK-180-pro). To demonstrate release behavior above and below the LCST of Soluplus (“OFF” and “ON” states, respectively), the wells were held at 35–37 °C (by keeping the orbital rocker inside a Binder KB-115 incubator) and occasionally cooled to 29 °C by removing wells from the incubator. Transition from high to low temperature (OFF→ON) was achieved quickly by shifting wells from the incubator to a precooled cooling plate (TE technology CP-121HT) (cooling profile of individual wells, measured using a wireless temperature transponder (BMDS TP-500), shown in Figure 4C), while transition from low to high temperature (ON→OFF) was achieved by placing wells into the prewarmed incubator (warming profile also shown in Figure 4C). In the OFF state, the wells had 0.3 mL drawn from them every 24 h. In the ON state, this amount was drawn every 20 min. As draws were done in triplicate, we added 0.9 mL of SBF at appropriate temperature following each time point to maintain constant volume. The cycle of warming and cooling employed for each study is described in detail in the results section. The remaining drug content within devices after completion of release studies was measured by end-point experiments in which the devices were soaked in 50 mL of an appropriate solvent (to facilitate full release) at 8 °C for at least 1 week; for Nile red and Curcumin, DMSO:SBF in a ratio of 9:1 or 4:1, respectively was used; for CXB, Methanol:SBF in a ratio of 9:1 was used. The quantity of remaining drug was summed with the amount of drug measured during the release experiments to determine the total initial drug loading of the composite devices. Methods for quantifying the payload present in wells using absorbance or fluorescence are described below; in all cases, the presence of Soluplus was found to have negligible effect on the absorbance/fluorescence readings of the payload. A Biotek Synergy H1 plate reader was used for all absorbance and fluorescence measurements.

### Nile Red

0.3 mL was drawn from the wells at appropriate times and combined with 2.7 mL DMSO. 0.3 mL of this mixture was then pipetted into a 96 well-plate well and quantified using fluorescence (excitation: 487 nm; emission: 528 nm).

### Curcumin

0.3 mL was drawn from the wells at appropriate times and combined with 1.2 mL DMSO. 0.3 mL of this mixture was then pipetted into a 96 well-plate well and quantified using absorbance at 420 nm.

### Celecoxib

0.3 mL was drawn from the wells at appropriate times and combined with 2.7 mL methanol. 0.3 mL of this mixture was then pipetted into a UV-transparent 96 well-plate well and quantified using absorbance at 260 nm.

### Temperature vs Release Rates

We placed CXB-laden composite devices into 10 mL wells of SBF. These devices were exposed to a range of temperatures from 26 to 37 °C and their corresponding CXB release rates measured. All experiments were done in triplicate. The resulting release rates were used to refine OFF and ON temperatures employed for subsequent *in vitro* cooling-triggered release experiments.

### *In Vitro* Cell Uptake Studies

Human Umbilical Vein Endothelial Cells (HUVECs; primary HUVECs acquired from ATCC) were exposed to solution (110 μL added to 1 mL of PBS covering cells) from release wells of OFF and ON state Nile red-laden composite devices for 30 min, then thoroughly washed with PBS. They were then observed using a confocal microscope with the 561 nm laser line used for excitation. As controls, unexposed HUVECs and HUVECs exposed to unladen Soluplus from blank devices were also observed.

Separately, GFP HUVECs (HUVECs expressing Green Fluorescent Protein) (Angio-proteomie) were cultured in 24-well plates with Nile red-laden composite devices. Composite devices were placed into wells containing GFP HUVECs cultured in 2.4 mL endothelial cell (EC) media. After incubation at 37 °C for 1 day, devices were briefly removed from wells, the media changed, and the cells imaged to visualize both GFP (indicating cell location) (ex/em: 482/524 nm) and Nile red uptake (ex/em: 542/593 nm) using an EVOS M5000. This process was repeated after a second day at 37 °C and again after a period of 40 min at room temperature plus 60 min warming back to 37 °C (total 100 min “cooling cycle” to assess cooling-triggered release and uptake of Nile red). Devices were then placed into a second well containing GFP HUVECs and the process described above repeated to assess Nile red uptake resulting from a second cooling cycle.

Additionally, human 1483 head and neck squamous cell carcinoma (HNSCC; chosen for their expression of COX-2) derived, characterized, and provided by Dr. Peter Sacks (New York University School of Dentistry, New York, NY) were acquired. These cells were exposed to solution from release wells of CXB- laden composite devices (110 μL from these wells added to 1 mL HBSS buffer covering the cells) and subsequently (30 min later) to 200 nM DMSO-dissolved FA similarly introduced. A Zeiss Axio Observer Z1 fluorescence microscope (Zeiss filter set 31; ex: 578 nm; em: 604 nm), was used to detect the extent of FA binding within the cells (after washing) and thereby indicate the extent to which successful CXB uptake had blocked potential FA binding sites. This competitive fluorescence assay is described in detail by Uddin et al.^46^ As controls, we also examined cells exposed only to FA (with no competition with CXB for binding sites) and cells exposed to FA and CXB dissolved in DMSO (a standard method of introducing CXB in similar experiments^46^).

### Cooling-Triggered Release in *Ex Vivo* Tissue

Slabs of tissue (∼1.5″ × 1.5″) from the hind legs of recently sacrificed adult, male Sprague–Dawley rats (within 1 h of death) were acquired. Composite devices were fabricated with payload consisting of both CXB and Nile red (to better imitate the release behavior of CXB while still using an easily quantifiable dye). These devices were then gently wrapped in warm, moist Kimwipes for 5 min (to wipe off any surface payload). The tissue slabs were then wrapped around the devices and left at various temperatures for up to 90 min. After this, the devices were removed from the tissue slabs and the Nile red within said slabs quantified using an IVIS with excitation and emission filters set to 560 and 620 nm, respectively.

### Cooling-Triggered Release *in Vivo*

A disposable 6 mm biopsy punch (Integra Lifesciences) was used to create 6 circular full-thickness wounds on the backs of two isoflurane-anesthetized, adult, male Sprague–Dawley rats (Figure 7B). These wounds were covered with a nonabsorbent silicone foam dressing (Mepilex, Molnlycke Health Care) held in place with an adhesive Tegaderm (3M) top layer and a Vetwrap (3M) bandage. After 48 h, the wounds were uncovered, and FA-laden composite devices (FA-laden Soluplus (0.001 w/w% FA) preparation similar to that of Nile red and Curcumin-laden formulations) shaped correctly for these wounds were placed into some wounds, while others were left empty as controls (see Figure 7B for positions of empty vs filled wounds). After implantation, wounds were covered (both treated and not) with Tegaderm, and one rat was left at body temperature for 40 min, while the other was cooled using a circulating chiller (Vevor Laboratory Chiller; −20C) such that its wounds experienced 24–26 °C for 40 min. The chiller was set to circulate a 2:1 glycerol: water mixture at 14 °C. To maintain a stable core body temperature during the cooling period, the rat was placed on a warming pad set to 38 °C (T/Pump, Stryker). The resulting temperature within the wounds was measured using a wireless temperature transponder (BMDS TP-500). Following the 40 min release period, the wounds were uncovered, and the FA-laden devices were removed from the wounds on both animals. Subsequently, both rats were sacrificed via CO_2_ inhalation, and the fluorescence signal of FA released from the composite devices was observed via IVIS (ex: 580 nm, em: 620 nm) (Figure 7B,C). All the procedures used in this study were performed in accordance with the guidelines approved by the Institutional Animal Care and Use Committee (IACUC) of Vanderbilt University; protocol number: M1900134.

## Results and Discussion

### Payload-Laden Soluplus Characterization

All Soluplus formulations we prepared (Table 1) were fluid, highly viscous (∼200,000 cP), and clear/lightly translucent at room temperature and nonflowing/gel-like and opaque at 35–37 °C (Figure 2A,B). Our formulation approach as described in the methods section is not expected to cause degradation of the loaded compounds or Soluplus. Specifically, heating the dry mixture of CXB and Soluplus at 130 °C is consistent with prior work, in which hot melt temperatures (130.5 and 165 °C) degraded neither CXB nor Soluplus.^31,50^ Furthermore, other previous work shows that both Soluplus and CXB experience no thermal mass loss below 200 °C;^51,52^ in fact, one study indicated that CXB experiences negligible mass loss when held at 170 °C for 1 h^51^ (compared to our method’s use of 130 °C for 30 min). Finally, when stored at 35–37 °C, formulations showed no signs of payload sedimentation over a week; all formulations were characterized or otherwise used within 5 days of production.

**Figure 2:**
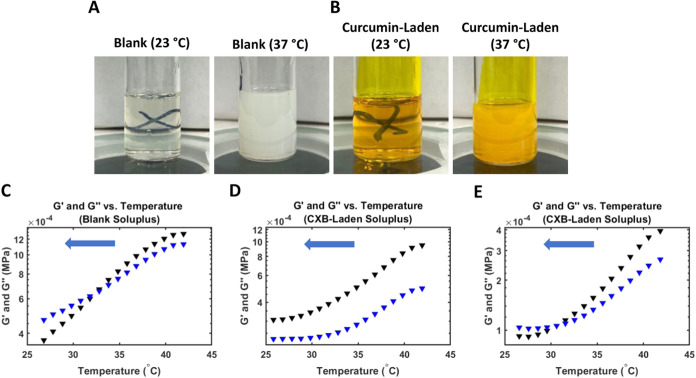
(A, B) Comparison of opacity of blank and curcumin-laden formulations below and above their LCSTs. (C–E) Downward temperature ramps showing the gel-to-sol transitions of blank and CXB-laden formulations. The blue arrows indicate cooling. Black triangles represent *G*′ and blue triangles represent *G*″. The point at which *G*′ falls below *G*″ is taken as the SP. (C) The temperature ramp for blank Soluplus (12.5 rad/s oscillation frequency); (D) the same for CXB-laden Soluplus using the same oscillation frequency; (E) the temperature ramp for CXB-laden Soluplus using 1.25 rad/s as the oscillation frequency.

We quantitatively characterized the LCST behavior of all formulations via temperature ramp experiments on an Ares G2 rheometer. These confirmed that, at elevated temperatures, the formulations exhibited gel-like behavior (storage modulus (*G*′) > loss modulus (*G*″)), while at lower temperatures, they exhibited more solution-like behavior (*G*″ > *G*′). Figure 2C–E show representative temperature ramps and Table 1 shows the transition temperatures; we define the solution point (SP) as the temperature at which *G*′ falls below *G*″ during a downward temperature ramp, and the gel point (GP) as the opposite during an upward temperature ramp. Note that CXB-laden Soluplus has a significantly different oscillation frequency response from the other 3 formulations, and so it was characterized using an oscillation frequency 10x smaller than that used for the rest (1.25 vs 12.5 rad/s). We report in Table 1 the GP and SP that were measured using the parameters found uniquely suitable for the CXB-laden formulation, but also show the corresponding temperature ramps obtained using both 12.5 and 1.25 rad/s (Figure 2D,2E, respectively). As described in prior work, Soluplus does not form a typical solid gel network even at elevated temperatures.^36,53^ Rather, it remains a viscous liquid material.^53^ However, for our purposes, we have chosen to describe its states as “gel-like” and “solution-like.” This approach is supported by existing literature.

It is known that viscoelastic liquids demonstrate frequency-dependent storage and loss moduli.^36,37,53^ Viscoelastic solids generally do not; however, as Soluplus remains a viscoelastic liquid even when “gel-like,” its storage and loss moduli remain frequency dependent throughout the temperature ramps employed herein. The apparent gel and solution points based on the previously described crossover of *G*′ and *G*″ are therefore also frequency dependent. For consistency, we therefore sought to use the same oscillation frequency for all samples studied, including the CXB-laden formulation. Other manuscripts have also used these crossover points to describe “gelation” of Soluplus, despite their frequency dependency, and found gel points similar to ours (∼36 °C for blank 30–40% Soluplus).^36^ However, as revealed by the frequency sweeps of blank and CXB-laden formulations (Figure S5 in supplementary data), using 12.5 rad/s for the latter would result in values showing gel-like behavior at all temperatures (Figure 2D). However, for the purpose of determining GP and SP, we required parameters that allowed for crossover of storage and loss moduli. As shown in Figure S5, our choice of 1.25 rad/s made this possible.

### Alginate- Soluplus Composite Device Characterization

Composite devices were fabricated as described in the experimental section. They had dimensions shown in Figure 3B and volume 0.36–0.4 mL. Figure 3A shows a schematic of the fabrication procedure and the final devices produced. Combining the alginate-LPEI with a Soluplus formulation, then stirring vigorously for 5 min resulted in a well-dispersed combination of the two fluids. This combination was always cast and cross-linked immediately after stirring.

**Figure 3:**
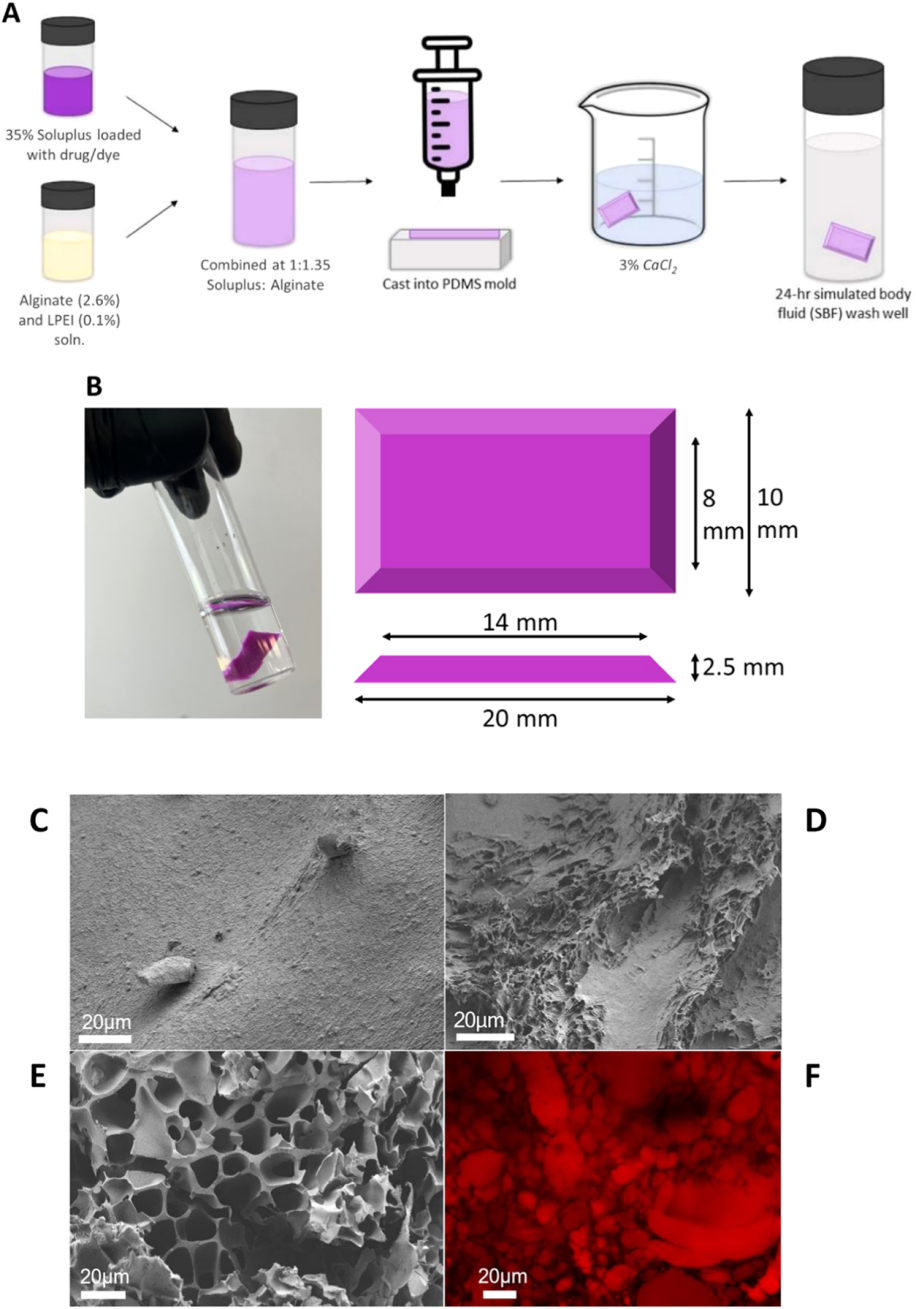
(A) Schematic showing fabrication of composite devices. (B) Dimensions and shape of composite devices. (C) Pure alginate device imaged via SEM. (D) An unvacated composite device imaged via SEM. (E) A partially vacated composite device imaged via SEM. (F) An unvacated composite device imaged via confocal microscopy.

Extensive microscale porosity was introduced to the alginate matrix by the presence of Soluplus domains (versus pure alginate). Figure 3C–F show this highly microporous structure in which alginate forms a matrix and Soluplus forms interspersed ∼20 μm diameter domains. Confocal microscopy examining a composite device (with Nile red-laden Soluplus) corroborates this matrix and domain structure (Figure 3F).

These alginate-Soluplus composite devices are meant to be temporary devices that degrade and/or erode safely without need for removal. Previous work has shown that Soluplus safely erodes/clears within a week.^54^ Additionally, alginate gel is known to degrade in physiological conditions.^55^ The geometry of our composites necessitates LPEI-reinforcement to maintain device integrity (>∼10 kPa complex modulus) for at least 2 weeks. Even with this reinforcement, composite devices lose around 80% of their complex modulus after 2 weeks (Supplementary data; Figure S6), indicating that, though the device did not suffer from premature disintegration, it is in the process of breakdown and resorption.

### Cooling-Triggered Payload Release from Composite Devices *In Vitro*

As shown in Figure 4A, CXB release rates (from composite devices held at constant temperature) are much higher at 26 and 29 °C than they are at 33, 35, and 37 °C; release at 26 and 29 °C was approximately 30x faster than at 35 °C and 75× faster than at 37 °C. The mean total mass of CXB in each of the 15 composite devices used for this experiment was 4.2 mg; this aligns well with the expected total mass of CXB per device based on device volume and CXB concentration (4.5 mg CXB before any loss during the wash step).

**Figure 4:**
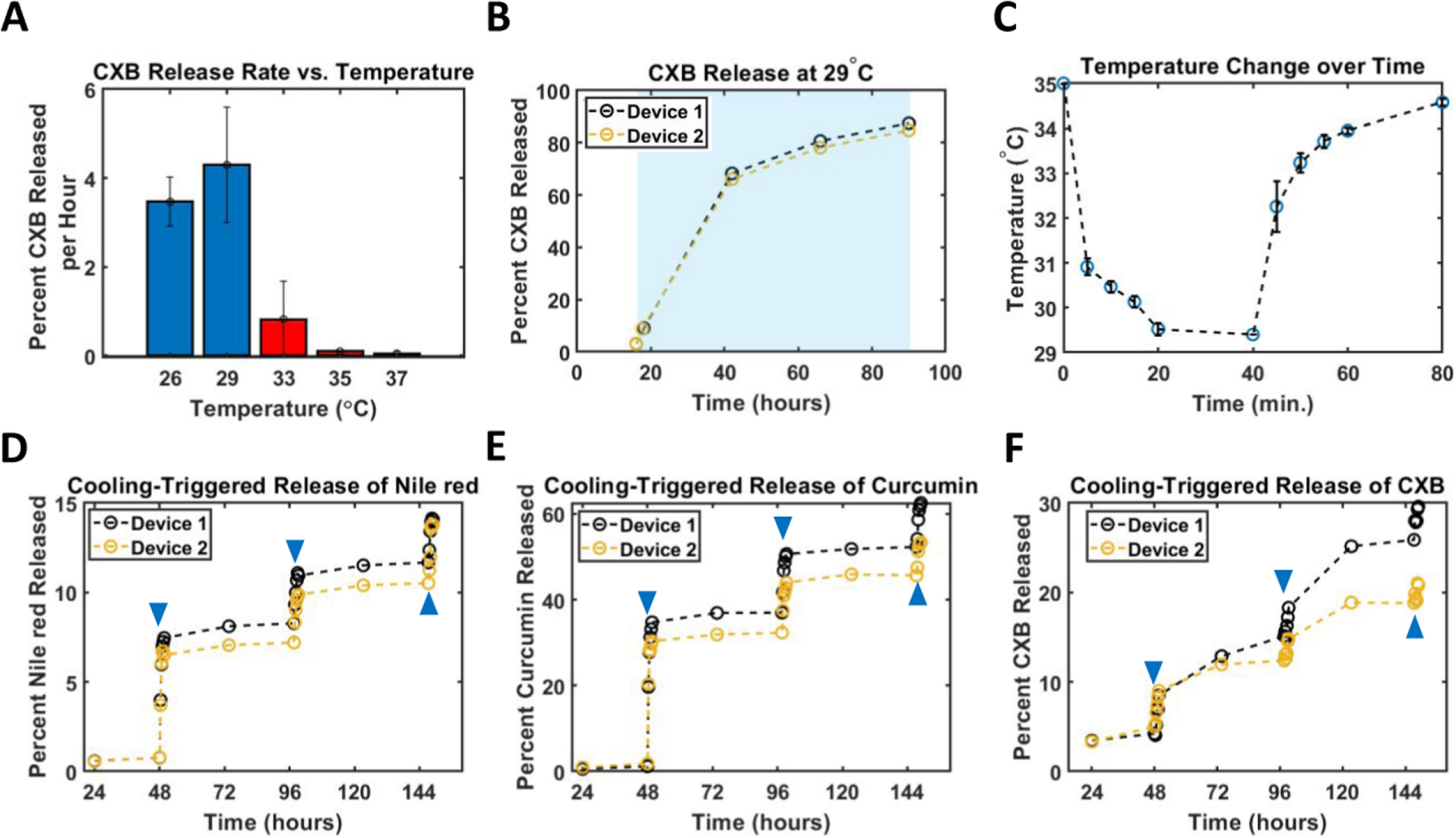
(A) Observed hourly release rates of CXB at selected temperatures. (B) Long-term cooling-triggered release of CXB. Nearly 90% of CXB payload is released within 3 days at 29 °C. (C) Temperature variation over time during (0–40 min) and after (after 40 min) active cooling; devices are placed back into warm incubator at 40 min. (D–F) Dynamic cooling-triggered release of (D) Nile red, (E) Curcumin, and (F) CXB from composite devices in 10 mL wells of SBF. Blue triangles represent application of cooling as shown in 4C.

We also tested longer-term low-temperature CXB release in SBF. CXB-laden devices were left at 29 °C for 72 h. During this period, they released almost 90% of their CXB payload (total 3.9 mg on average). This experiment was done in triplicate and its results are shown in Figure 4B.

To test cooling-triggered release cycling (varying temperature over time), devices were placed into 10 mL wells of SBF. These wells were then subjected to a series of temperature changes over 6 days, as shown in Figure 4D–F. We used 35 and 37 °C as the high (OFF) temperature for CXB-laden devices and Nile red/Curcumin-laden devices, respectively, and 29 °C as the low (ON) one. Devices were left at their OFF temperatures for 2-day periods, after which they were actively cooled for 40 min. The effect of cooling on payload release was observed for 100 min. The change in temperature experienced by the devices over time during and after cooling- as measured by wireless transponder- is shown in Figure 4C. After each two-day period and subsequent cooling cycle, the devices were moved into new 10 mL SBF wells. This was done to ensure that CXB release during the second and third cooling cycles would not be artificially diminished by a reduced concentration gradient resulting from previous release cycles. Preliminary experiments showed that shifting wells after each cooling cycle does not significantly affect release rates in the subsequent OFF states.

Prior work^23^ has shown that safe cooling of human skin using an icepack can result in temperatures below 16 °C subcutaneously or ∼28 °C intramuscularly (1 cm below the fat layer) within 20 min. The same publications also show that the subcutaneous layer can be assumed to remain above 31 °C in reasonable ambient conditions without cooling, while the intramuscular region can be assumed to maintain 35 °C. The temperature spread used in our cooling-triggering experiments therefore closely imitates the spread that would be observed intramuscularly (1 cm below subcutaneous fat) both triggered by cooling and untriggered. Note that Nile red and Curcumin-laden devices had to be tested with a higher OFF temperature of 37 °C, as at 35 °C they experienced higher leakage rates. Also, we acknowledge that unusually cold ambient conditions combined with inadequate skin covering could result in subcutaneous and intramuscular temperatures lower than those we have assumed. However, as shown by Webb,^56^ for a patient’s intramuscular region to fall below 31 °C, they would need to be exposed to conditions that would induce “strong shivering.” We expect most patients would avoid such conditions and that exposure to such environments would be abnormal.

The three CXB-laden devices used for this experiment had a mean CXB loading of 3.9 mg. Hence, by releasing ∼5% of this total during the first and second cooling cycles, these devices released ∼200 μg of CXB each time (similar to the 200 μg CXB applied to counteract formalin-induced hyperalgesia in rats in prior work^15^). The third cooling cycle (and to a lesser extent, the second) produced a diminished rate of CXB release; this also occurred with Nile red and Curcumin-laden devices. Such behavior makes sense given that allowing release of the majority of Soluplus domains near the surface of a composite device leaves only deeper domains which have a longer, more tortuous path to release upon subsequent triggering. However, even the third cycle resulted in 115–160 μg of CXB release; this remains within the same order of magnitude used to demonstrate CXB-based analgesia in prior work. Examples include work in which induced hyperalgesia in adult rats is counteracted by as little as 200–250 μg of CXB.^15,57^ This range modestly overlaps with the mass of CXB released from our devices during cooling cycles. Additionally, if necessary, the amount of CXB released could be increased by lengthening the period of active cooling and/or employing more than one consecutive cooling events. Comparison to dosing previously used in rats makes sense given the size of our composite devices (0.36–0.4 mL). For eventual application in a clinical setting, said devices would need to be scaled up.

As shown in Figure 4, both Curcumin and CXB demonstrated excellent cooling-triggered release characteristics (from our composite devices); they both showed low leakage rates in the OFF state (generally under 5%/day, except following the second release cycle of CXB) and high ON/OFF ratios (>∼30x). These drugs are both highly hydrophobic weak acids with high p*K*_a_ values (Curcumin: p*K*_a_= 7.4, 9.6, and 10.9; CXB: p*K*_a_ = ∼11). We anticipate that other similar drugs should demonstrate similar compatibility with the Soluplus-alginate composite platform. This hypothesis is further supported by experiments on the Indapamide and Estradiol, both of which are hydrophobic weak acids but with different p*K*_a_ values of 8.8 and 10.7, respectively (Figure S7).

### Solubilization of Released Payloads

We also examined the form in which payloads were released from the composite devices. First, we determined the solubilization states of the total mass of drug/dye released from these devices over 2 days OFF plus a cooling cycle (100 min total, of which cooling to 29 °C was applied for 40 min). Total payloads released in this period were 6, 910, and 400 μg, for Nile red, Curcumin, and CXB, respectively. We found that Curcumin and CXB both remained highly solubilized after release (∼90 and 70%, respectively), while Nile red did not; this is shown in Figure 5A. As the payloads were released with Soluplus, we assumed the observed high level of solubilization was attributable to the well-known micellization behavior.^37^ To test this, we employed DLS (Figure 5B) to observe wells with payloads released over the time period and temperatures described above. The results showed particle size distributions centered around ∼60 nm at room temperature; this is consistent with the presence of Soluplus micelles.^37^ Increasing the temperature of the tested solutions to 37 °C resulted in increased particle hydrodynamic diameter; this is also consistent with the thermothickening behavior of Soluplus, which is generally attributed to clumping of micelles to form larger clusters. We also directly observed micelles using TEM. As shown in Figure 5C, TEM confirms that micelles (both blank and CXB-laden) are present and ∼60 nm in diameter.

**Figure 5:**
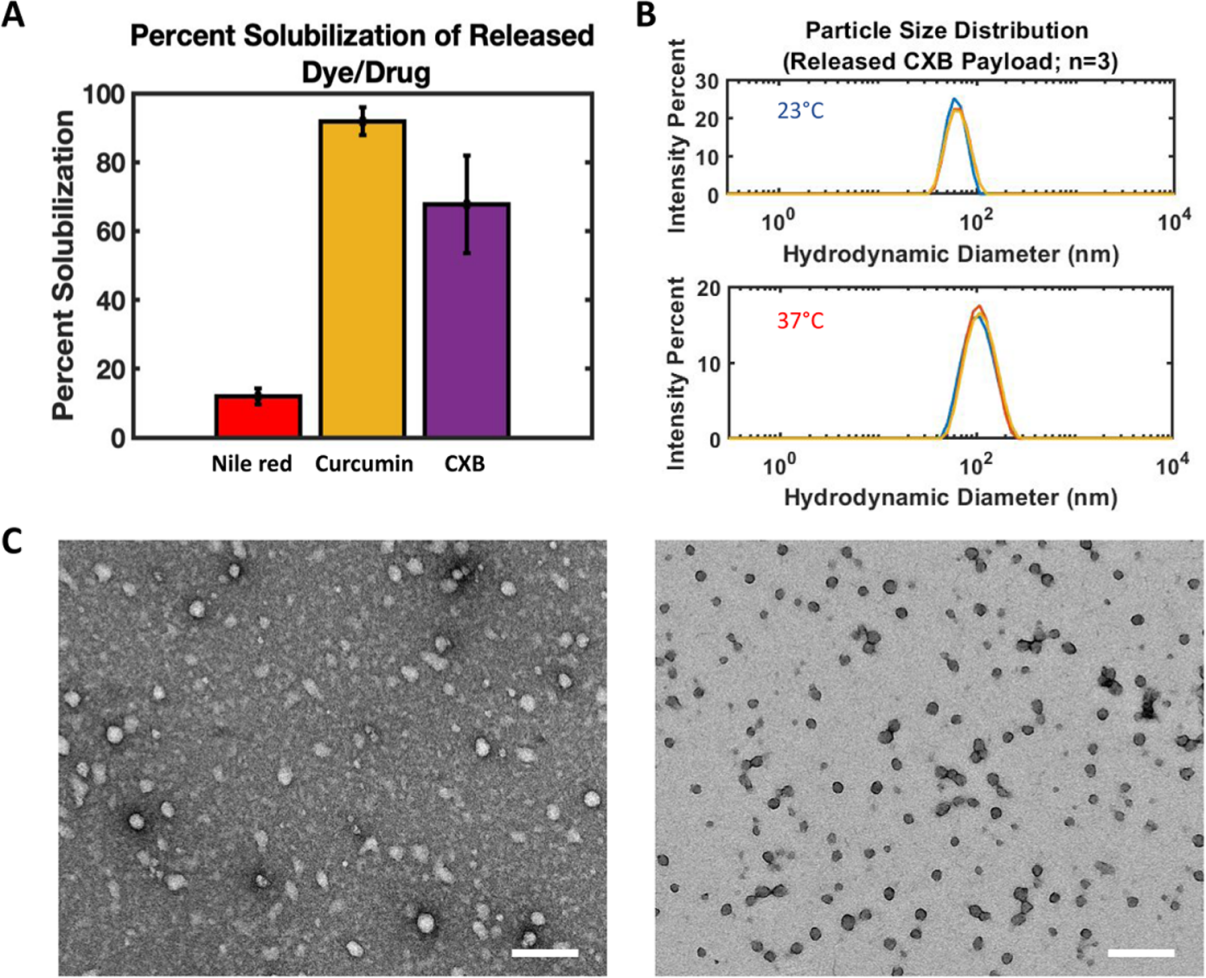
(A) Percent solubilization of Nile red, Curcumin, and CXB in wells after release from composite devices. (B) Hydrodynamic diameter of CXB-laden Soluplus micelles released from composite devices. Top = micelles at 23 °C and bottom = micelles at 37 °C. (C) TEM images showing presence of blank (left) and CXB-laden (right) micelles in composite devices release wells. Scale bar = 200 nm.

As noted above, Soluplus’ ability to solubilize poorly soluble drugs is well-known. Various authors^31–35^ have shown that it remains stable and significantly enhances solubility of these drugs even at high drug loadings. For example, 20% CXB in Soluplus was shown to constitute a stable formulation, with solubility enhanced by a factor of 2 even after a year of storage.^31^ While we were able to confirm this drug loading capacity in preliminary studies, we also found that higher drug percentages reduced the magnitude of thermothickening effect relied on for cooling-triggered drug delivery; we therefore opted to use a more moderate drug loading (8w_CXB_/w_Soluplus_%).

Given the observed solubilization of both Curcumin (over 100-fold increase over the 0.6 μg/mL previously reported^58^) and CXB (about 6-fold increase in solubilization over the 5 μg/mL previously reported^59^) shown in Figure 5A and the observation of Soluplus micelles via DLS and TEM in Figure 5B,5C, respectively, we hypothesize that the dye/drug payloads are released from composite devices in Soluplus micelles. This is also supported by the known ability of Soluplus to form micelles that can be loaded with and support solubilization of hydrophobic drugs with low aqueous solubility.^31–35^ Our preliminary observation that hydrophilic drugs rapidly diffuse into the surrounding aqueous solution regardless of temperature further supports that integration of hydrophobic drugs into Soluplus micelles is key to the triggerable release mechanism. Additionally, as discussed above and in the supplementary data (Figure S7), the hydrophobic drugs with the best cooling-triggered release characteristics are those whose aqueous solubility is the lowest at physiological pH.

### *In Vitro* Cellular Uptake of Released Payloads

Soluplus, a known drug excipient, has been shown to be effective at delivering hydrophobic drugs to cells. To confirm the excipient was delivering the payloads, we tested how well Nile red released during cooling from composite devices was absorbed by HUVECs. As a preliminary study, HUVECs that were exposed to Nile red released from composite devices (incubated separately), then washed thoroughly, showed clear, strong red fluorescence implying uptake of the dye (Figure S2A). Controls showed no comparable fluorescence.

We also coincubated composite devices and GFP HUVECs; Figure 6A shows the resulting green and red channels after a cooling cycle. Figure 6B shows the calculated rate of Nile red uptake under different temperature conditions. The red Nile red signal is normalized by the area occupied by GFP HUVECs (the green signal) in each image; we used ImageJ to define a mask that separated the green cell-covered regions from the background, allowing us to also separate red signal attributable to cells from background red signal. Note that cells were washed to ensure that only absorbed Nile red would remain during imaging. Preliminary studies showed that after washing (media change), the Nile red present in HUVECs quickly equilibrates to the Nile red concentration of the newly added media (Figure S2B). We therefore always imaged cells immediately after changing the media.

**Figure 6:**
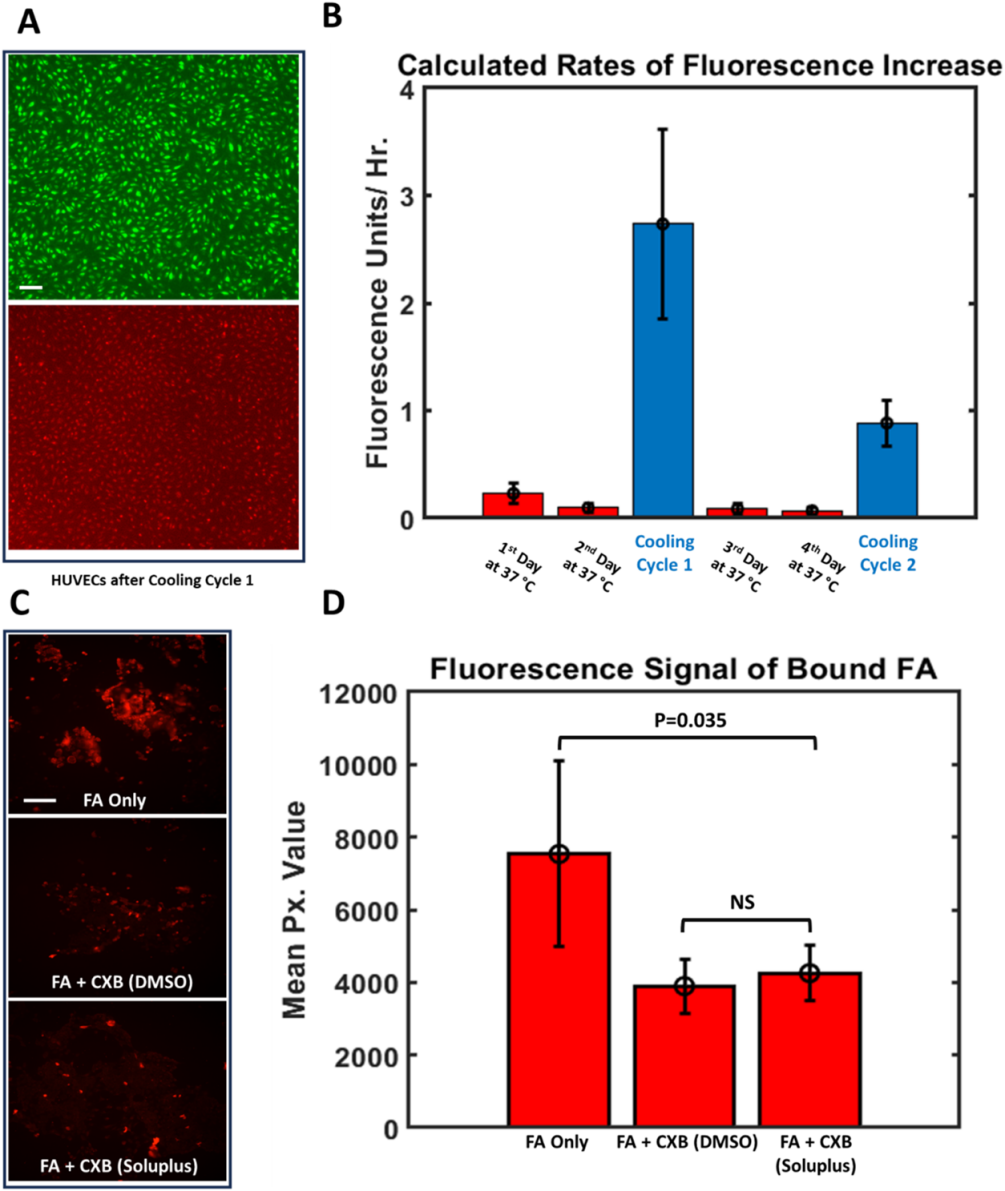
(A) GFP HUVECs coincubated with composite devices showed significant Nile red uptake. The green channel (top) was used to create a mask localizing cell location, while the red channel (bottom) was used to quantify Nile red uptake. (B) Calculated rates of Nile red uptake into HUVECs coincubated with composite devices under different temperature conditions (*n* = 5). (C) Competitive fluorescence assay showing uptake of CXB released from composite devices. Loss of fluorescence when 5 μM CXB released from composite devices is added prior to FA indicates high levels of cellular uptake: top shows HNSCCs exposed to FA only; middle shows HNSCCs pre-exposed to CXB dissolved in DMSO; bottom shows HNSCCs pre-exposed to CXB released from composite devices. Both scale bars represent 200 μm. (D) Quantitative comparison (*n* = 3 for CXB pre-exposed groups; *n* = 4 for “FA Only” group) of FA fluorescence with and without pre-exposure to CXB (from composite devices and dissolved in DMSO). Pre-exposure to CXB released from composite devices caused a statistically significant drop (*p* = 0.035 on log-transformed data using a one-sided Welch’s *t* test) in fluorescence compared to the FA Only group.

While cells can be seen to take up Nile red from composite devices during the OFF-state periods (37 °C), the uptake is shown to be over 10 times as fast during the subsequent 100 min cooling cycles (40 min at room temperature, followed by 60 min re-equilibrating to 37 °C).

Similarly, we tested the uptake of CXB released from composite devices using 1483 HNSCC cells. These cells were chosen for their expression of COX-2, a binding site for NSAIDs such as CXB and dye-tagged NSAIDs like FA (5-ROX-tagged Indomethacin). When 1483 cells were exposed to 200 nM FA only, then washed thoroughly, they showed strong fluorescence attributable to uptake and binding of FA. However, when the cells were pre-exposed to 5 μM CXB released from CXB-laden composite devices, they showed a marked drop in subsequent FA binding (detectable by a reduction in postwash fluorescence). This indicates that CXB released from our devices can enter and effectively bind to COX-2 binding sites within the cells and thereby block FA from binding to the same sites. For further comparison, we also showed that 5 μM CXB (with CXB in free solution in DMSO, the conventional method used for this competitive fluorescence assay^46^) introduced to the cells did not cause a larger drop in fluorescence than that seen using CXB delivered from our devices (Figure 6C,6D).

### Cooling-Triggered Payload Release in *Ex Vivo* Tissue

To test if cooling-triggered release (and lack thereof at body temperature) within tissue imitates that observed *in vitro*, we studied release within tissue of recently sacrificed Sprague–Dawley rats (Figure 7A). Experiments were conducted immediately upon sacrifice. To minimize the effect of surface payload simply “rubbing off” onto tissue even at OFF temperatures, we gently wiped composite devices with moist Kimwipes for 5 min prior to starting the experiment. This wiping step was incorporated into *in vitro* cooling-triggered release shown in Figure 4 for consistency.

**Figure 7:**
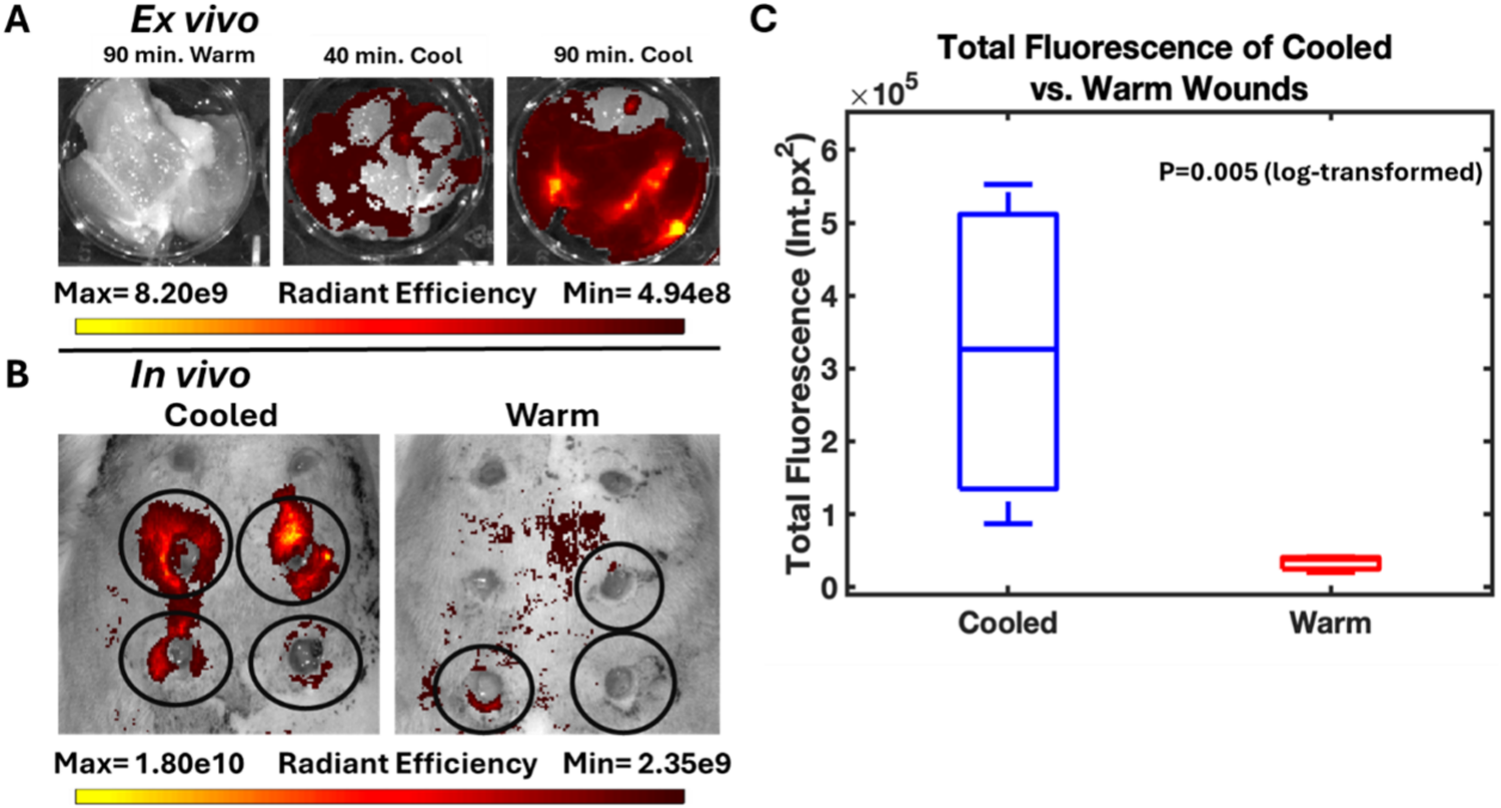
(A) Tissue exposed to composite devices at 37 °C for 90 min (left), at 23 °C for 40 min (center), and at 23 °C for 90 min (right). (B) IVIS fluorescence images of wounds exposed to FA-laden cooling-triggered devices at 24–26 °C (“cooled”; left) and body temperature (“warm”; right). Black circles show which wounds had devices in them; the remainder were left empty. (C) Background-corrected total fluorescence of cooled vs warm wounds. The difference between cooled and warm wounds is statistically significant (*p* = 0.005 on log-transformed data using a one-sided Welch’s *t* test).

As noted in the Experimental Section, the composite devices used for *ex vivo* tissue release experiments contained a mixture of CXB and Nile red (with the Nile red and CXB portions matching those used in their respective individual formulations). This was done to ensure that the rheometric characteristics of the device matched those of other CXB devices closely, while still allowing for a fluorescence-based reading. Fluorescence readings taken via IVIS showed that after 90 min at 37 °C, very little Nile red is absorbed by the tissue in contact with cooling-triggered devices. Simultaneous experiments showed much greater absorption of Nile red at 23 °C after both 40 and 90 min. 90 min was selected as a relatively long time period while not being so long that the tissue starts to degrade. 40 min was chosen as it mimics the *in vitro* release period.

### Cooling-Triggered Payload Release in *In Vivo* Tissue

Finally, to explore cooling-triggered release from devices in living tissue, appropriately shaped (5 mm diameter; 2 mm high) composite devices were placed inside excisional skin wounds on the backs of Sprague–Dawley rats for 40 min and either allowed to stay at body temperature or cooled to 24–26 °C. Figure 7B below shows the positions of the wounds (both empty control wounds [not circled], and wounds filled with a composite device [circled]) superimposed on IVIS images representing results of the cooling-triggered release experiment. Figure 7C shows the total fluorescence intensity measured for each wound, accounting for background signal. The method used to determine background-corrected total fluorescence can be found in the Supplemental Data (Figure S3). Devices in cooled wounds released significantly more FA than those in warm wounds.

## Conclusion

We have demonstrated hydrogel composite devices composed of a drug-laden thermoresponsive (LCST) polymer hydrogel constrained by a nonthermoresponsive matrix hydrogel. These composite devices have been morphologically characterized and their thermoresponsiveness quantified both in terms of change in modulus with temperature and in terms of change in drug release rates with temperature. We have also demonstrated cooling-triggered release of CXB and related compounds from these devices *in vitro* and *in vivo*.

These results indicate that this implantable, macroscopic platform can be used to achieve cooling-triggered, local NSAID release. Such a platform could serve as an alternative to oral administration of drugs like opioids, primarily in cases of localized pain and inflammation (such as postoperative pain). Going forward, we envision cooling being used more broadly as an external stimulus for targeted, patient-controlled pain relief. Unlike most other methods for external triggering of drug release, cooling does not require any energy input or complex apparatus; simple application of an ice pack to the region of interest (a procedure familiar to patients) could be sufficient to trigger release of a payload sequestered within an implanted device.

The device design described herein allows for variation of the specific thermoresponsive and nonthermoresponsive components as appropriate for the desired application. Additionally, we view this approach as being highly versatile (showing successful triggered release for CXB, Curcumin, and [with reduced ON/OFF ratio] Nile Red) and anticipate various other hydrophobic drugs could be compatible with this concept. Lastly, as Soluplus continues to be explored for its ability to solubilize and improve efficacy of various drugs, our use of this excipient places this platform in an excellent place to add stimulus-responsiveness to the other formulations currently under development.

## Supplementary Material



## Notes

Chemical structures of species used, preliminary study showing Nile red uptake into HUVECs exposed to the release wells of separately incubated composite devices, preliminary study demonstrating equilibration of HUVECs Nile red signal to newly changed media (with no Nile red), the method used to determine background-corrected Fluorocoxib-A signal in in vivo wounds, TGA data, Soluplus rheometry frequency-dependency data, alginate degradation data, and cooling-triggered release of Indapamide and Estradiol (PDF)
