# Supplementary material for: Cooling-Triggered Release of Celecoxib from Implantable Alginate-Soluplus Composite Devices
Source: ACS Biomater Sci Eng. 2025 Aug 25;11(9):5413–25. doi: 10.1021/acsbiomaterials.5c00867 (PMC12421509; doi:10.1021/acsbiomaterials.5c00867)
Supplement: Supplementary file 1 [file ab5c00867_si_001.pdf]

Electronic Supplementary Information: Cooling-Triggered Release of Celecoxib from Implantable Alginate-Soluplus Composite Devices.

Authors: Romario Lobban[1], Michael Carroll[2], Victoria Vest[1], Josh T. McCune[3], Sarah Hall[3], Fang Yu[3], Md. Jashim Uddin[3,4], Lawrence J. Marnett[4,5,6], Craig Duvall[3,7,8], and Leon M. Bellan[1,3]

1: Vanderbilt University Mechanical Engineering Department

2: Vanderbilt University Division of General Engineering

3: Vanderbilt University Biomedical Engineering Department

4: Vanderbilt University School of Medicine, Department of Biochemistry

5: Vanderbilt University School of Medicine, Department of Chemistry

6: Vanderbilt University School of Medicine, Department of Pharmacology

7: Vanderbilt University Chemical and Biomolecular Engineering Department

8: Vanderbilt University Department of Ophthalmology and Visual Sciences

Chemical Structures of Compounds Used

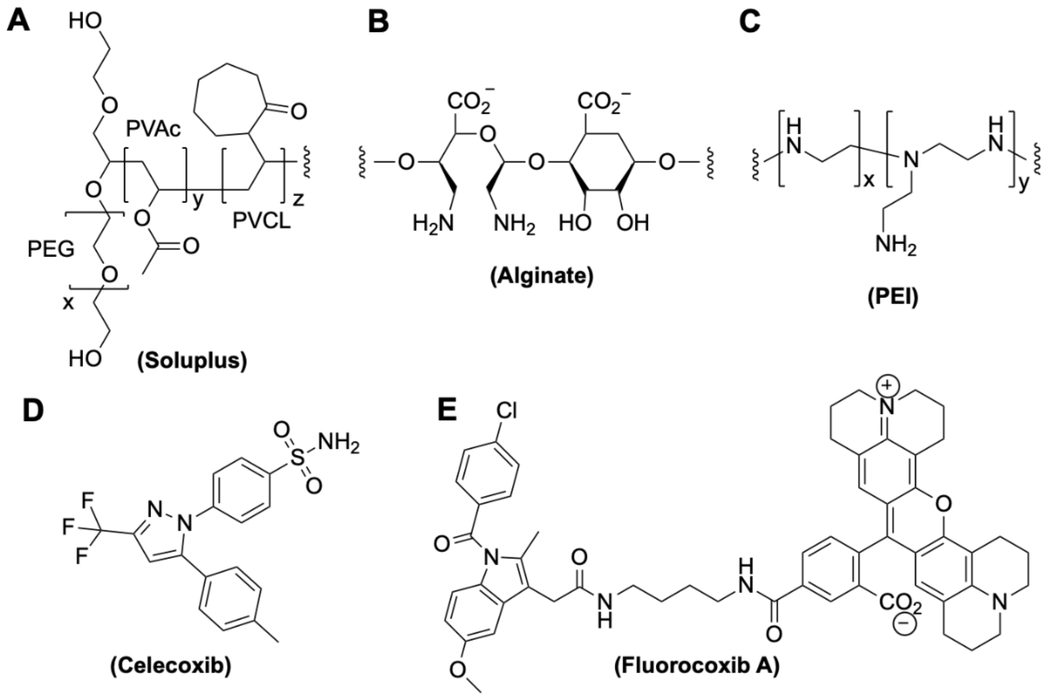

Fig. S1. Chemical structures of compounds used.

HUVECs Nile red Uptake and Nile red Signal Decay

Figure S2A below shows Nile red uptake into HUVECs from composite devices incubated separately. The cells show significantly higher red signal when exposed to the release well contents of "ON" state composite devices.

Figure S2B below shows the decay of Nile red signal localized to GFP-HUVECs after the media covering said cells was changed to Nile red-free media. As described in the results section, the Nile red signal was determined by using the green signal to create a mask that defined which regions of red signal were attributable to cells vs. the background. As shown in the figure, after 80-100 minutes, the HUVECs' Nile red content has mostly equilibrated to the Nile red concentration (0%) in the newly added media.

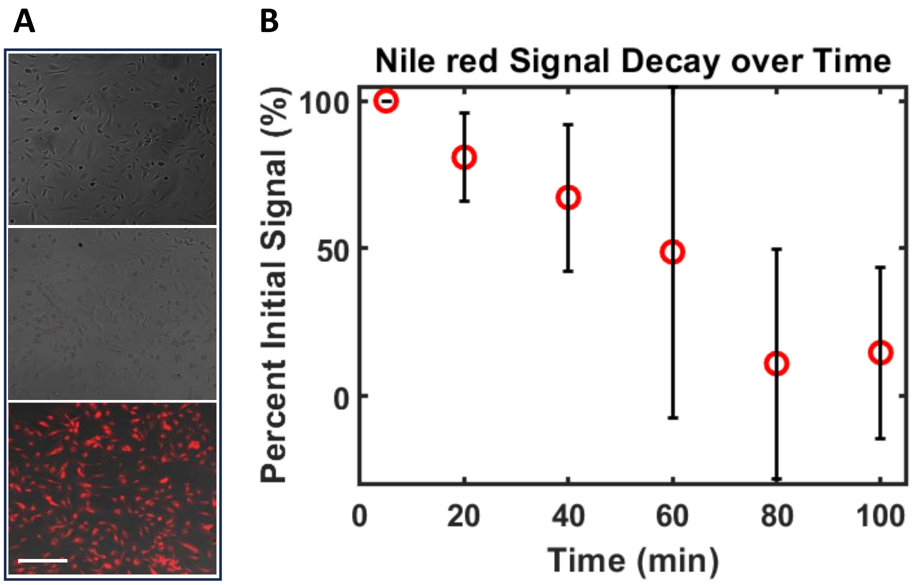

Fig. S2. A.) HUVECs exposed to Nile red laden Soluplus released from composite devices show high levels of Nile red uptake: top shows HUVECs with no Nile red added; middle shows HUVECS exposed to OFF state of Nile red-laden composite device; bottom shows HUVECs exposed to ON state of Nile red-laden composite device. Scale bar represents 200 microns. **B.)** Decay of Nile red signal attributable to GFP-HUVECs over time (n=6) after replacement of Nile red-rich media with Nile red-free media.

***In vivo* Wound Fluorescence Quantification**

Figure S3A below shows grayscale IVIS fluorescence images (ex: 580 nm, em: 620 nm) of 2 Sprague Dawley rats (post-sacrifice). Previously, the rats had 5 mm diameter, 2 mm tall, FA-laden (0.001 w/v% FA) cooling-triggered devices placed inside 2-day old wounds on their backs. The top image shows devices placed on a rat and kept at 24-26 °C for 40 minutes. The bottom image shows devices placed on a rat and kept at ~37 °C for 40 minutes. The IVIS images were captured after the cooling-triggered devices were removed. Wounds circled in red were left empty and used as blanks to help determine background fluorescence.

To quantify fluorocoxib A (FA) release, we first imported the grayscale IVIS images into ImageJ for analysis. We verified that pixel intensity in these images scales linearly with radiant efficiency, with a pixel value of 0 corresponding directly to a radiant efficiency of 0. Given the roughly circular nature of the wounds in our samples, we employed a spatially resolved approach by examining the distribution of mean pixel intensity as a function of distance from the wound center. Specifically, we identified the central point of each wound and measured the average pixel intensity along a series of concentric circles drawn at increasing radii from this center. This method enabled us to plot the radial profile of FA release, yielding a detailed mapping of pixel intensity—and thus radiant efficiency—at various radial positions. By analyzing the average pixel values over these concentric regions, we obtained quantitative insights into the spatial distribution of FA release across the wound area. The 5 blank wounds were used to estimate average background fluorescence both within wounds and on the boundaries of wounds (transition between wound background and the higher intensity skin background). This was combined with the background fluorescence of non-wound skin at ∞ surrounding each wound (mean intensity in a 180-200-pixel square [depending on available space surrounding each wound] centered on the wound minus an 80-pixel radius circle also centered on the wound) to estimate an appropriate background correction.

Figure S3B below shows example plots of fluorescence intensity vs. distance from wound center. The black lines represent raw mean pixel value data. The broken blue lines represent the estimated background correction. The orange lines represent the corrected mean pixel value data.

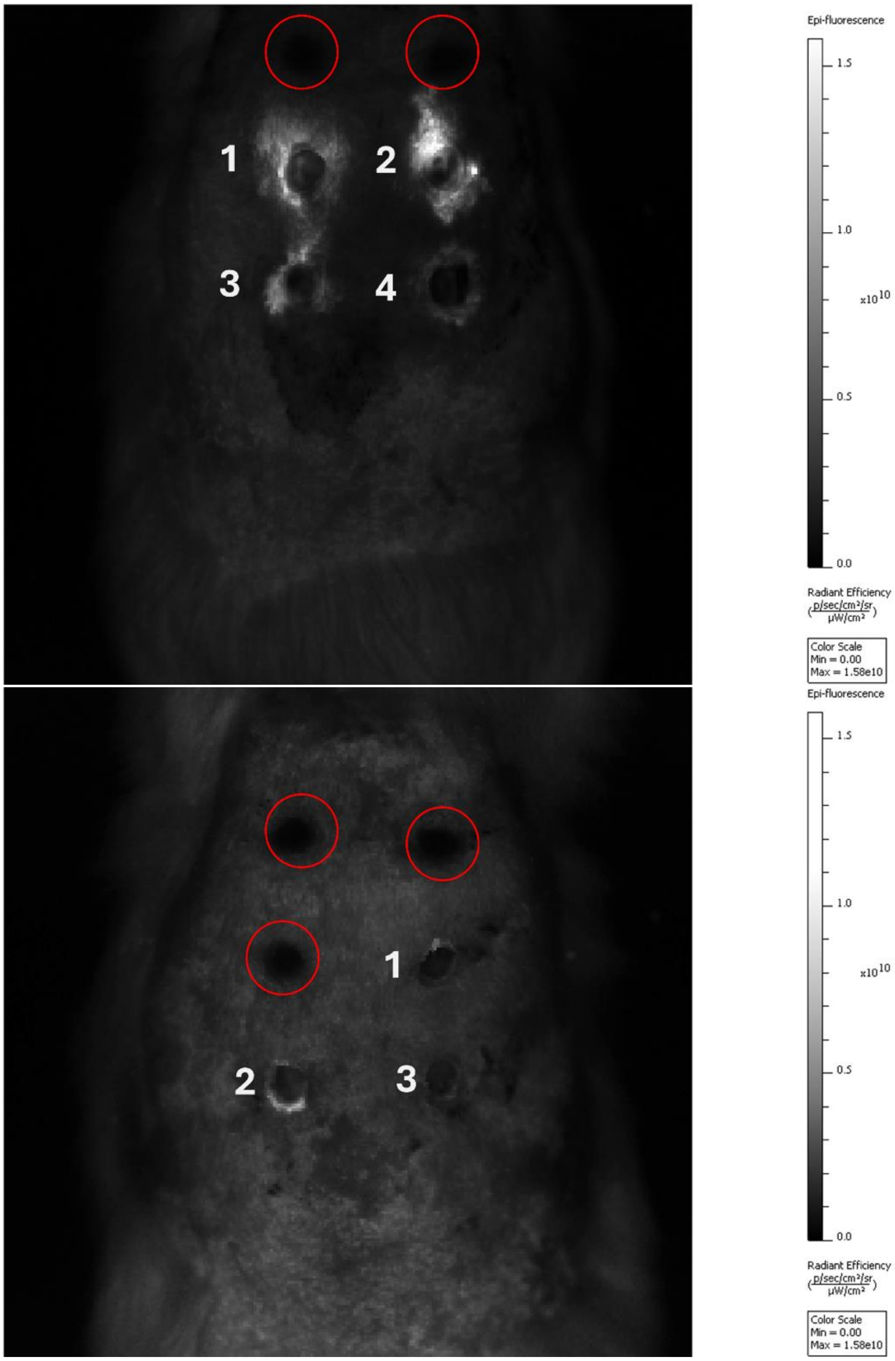

1
2
3
4
Epi-fluorescence
1.5
1.0
x10^10
0.5
0.0
Radiant Efficiency
(p/sec/cm²/sr / μW/cm²)
Color Scale
Min = 0.00
Max = 1.58e10
1
2
3
Epi-fluorescence
1.5
1.0
x10^10
0.5
0.0
Radiant Efficiency
(p/sec/cm²/sr / μW/cm²)
Color Scale
Min = 0.00
Max = 1.58e10

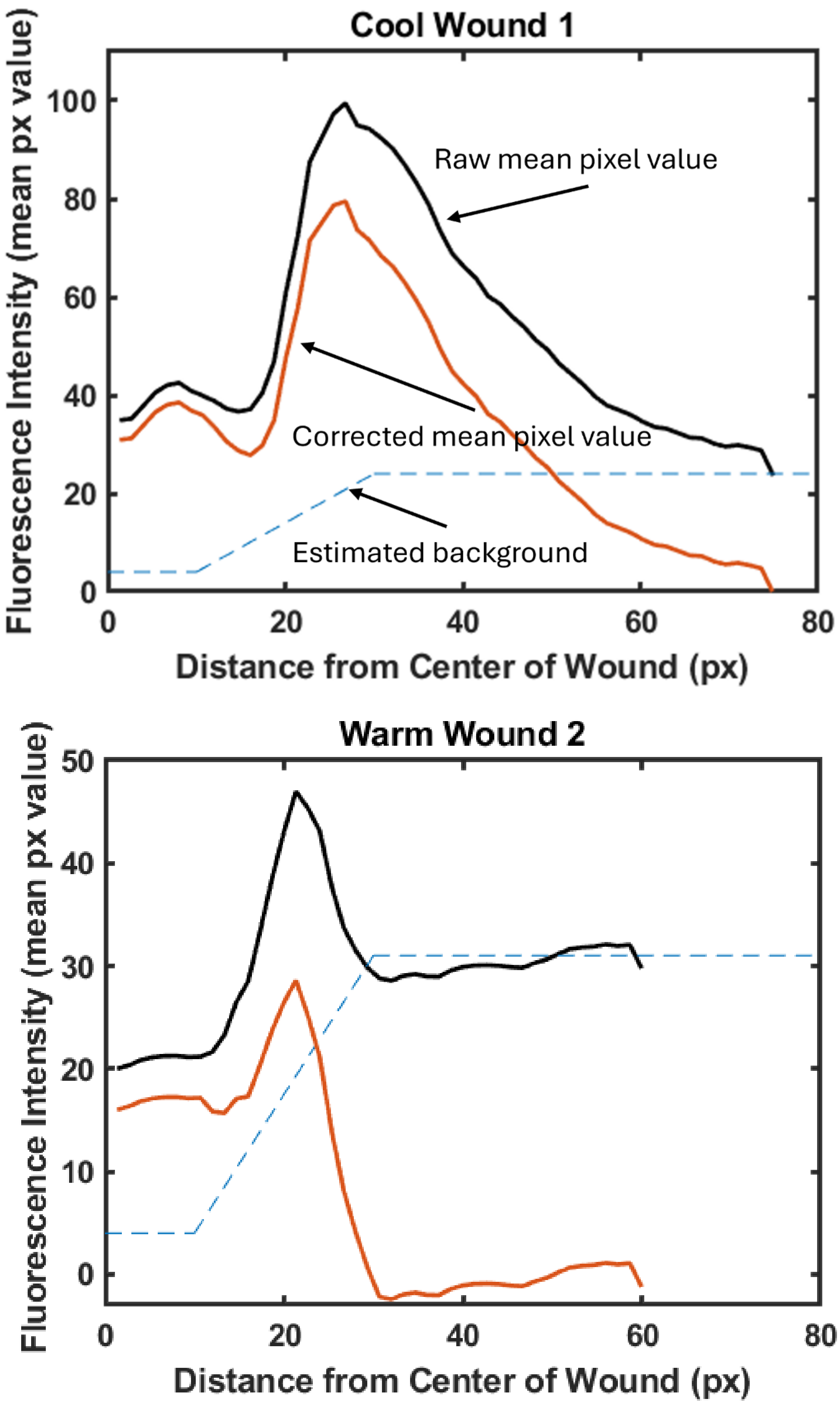

Fig. S3. A. Grayscale IVIS fluorescence images of wounds exposed to FA-laden cooling-triggered devices at 24-26°C ("cooled"; top) and body temperature ("warm"; bottom). Red circles show which wounds were left empty. Wounds exposed to devices are

numbered. **B.** Fluorescence intensity vs. distance from the center of a cooled wound (top) and a warm wound (bottom). The black lines represent raw mean pixel value data. The broken blue lines represent the estimated background correction. The orange lines represent the corrected mean pixel value data (raw mean pixel data minus estimated background).

The orange lines were integrated radially to determine the background-corrected total fluorescence (in Int.px^2) over the regions of interest. These results are shown in fig. 7 in the main manuscript.

Thermogravimetric Analysis of Soluplus, CXB, and the Combination thereof

Figure S4 below shows thermogravimetric analysis (TGA) data confirming the lack of thermal degradation of Soluplus (S4A), CXB-laden Soluplus (S4B), and CXB (S4C) at the temperatures described in this manuscript (up to 130°C).

All TGA was performed on an Instrument Specialists Incorporated TGA 1000 with an aluminum crucible. Tests were performed under a Nitrogen atmosphere with a flow rate of 100 ml/minute. Approximately 2 mg of CXB was tested from 25°C to 600°C, while approximately 10 mg of Soluplus and CXB-laden Soluplus were tested from 25°C to 900°C. Finally, all TGA was carried out at a heating rate of 20°C/minute. As shown, thermal mass loss onset occurred at 323°C for both the Soluplus and CXB-laden Soluplus samples, whereas mass loss onset occurred at 307°C for the CXB sample. Both the blank Soluplus sample and CXB-laden Soluplus samples experienced loss of moisture early in the temperature ramp; this is a known phenomenon reported by others[1].

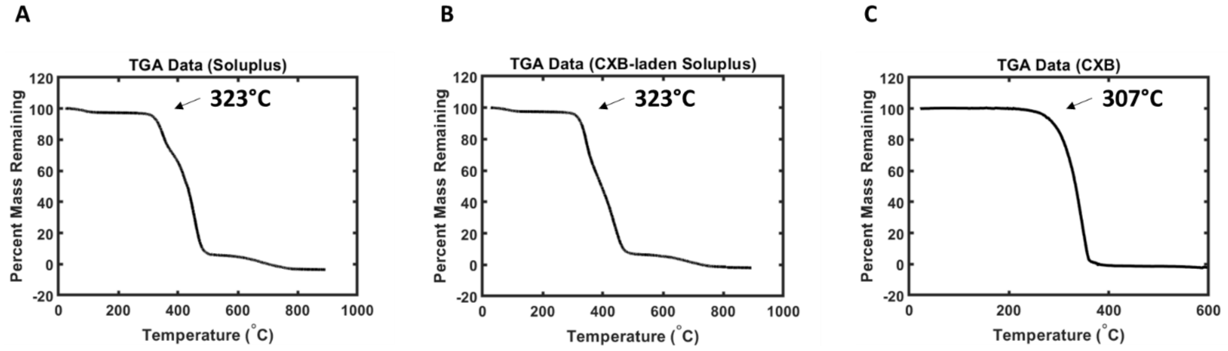

Fig. S4. TGA data for A.) Soluplus, B.) CXB-laden Soluplus, and C.) CXB. All mass loss onsets occurred far beyond the temperatures used in our manuscript.

Frequency Dependency of Blank and CXB-laden Soluplus Rheometry

Figure S5 below shows frequency sweeps of blank and CXB-laden Soluplus formulations at 23°C and 35°C, respectively. Frequency sweeps were carried out on an Ares G2 rheometer with 20mm parallel plate geometry, a 1 mm gap, and constant stress of 5 Pa.

As shown, when 12.5 rad/s is chosen, both formulations exhibit gel-like behavior (G'> G'') at temperatures greater than or equal to ~35°C. However, at 23°C, while the blank formulation (and Nile red and Curcumin-laden formulations) seems to exhibit solution-like behavior (G'' >G'), the CXB-laden formulation continues to demonstrate gel-like behavior (G'> G'').

When 1.25 rad/s is chosen, both formulations exhibit solution-like behavior at 23°C (G''> G'). However, at temperatures greater than or equal to ~ 35°C, only the CXB-laden formulation transitions to the gel-like state (G'> G'').

12.5 rad/s provides usable crossover points for more of our formulations, is more similar to frequencies used in previous work[2,3] that also sought to quantify the behavior of blank Soluplus, and provides crossover points similar to those found previously[2,3] using similar rheometric parameters. We therefore chose to use it for measurement of all formulations (including CXB-laden formulations). However, for the sake of finding a GP and SP for the CXB-laden formulation, we also characterized the CXB-laden formulation using 1.25 rad/s.

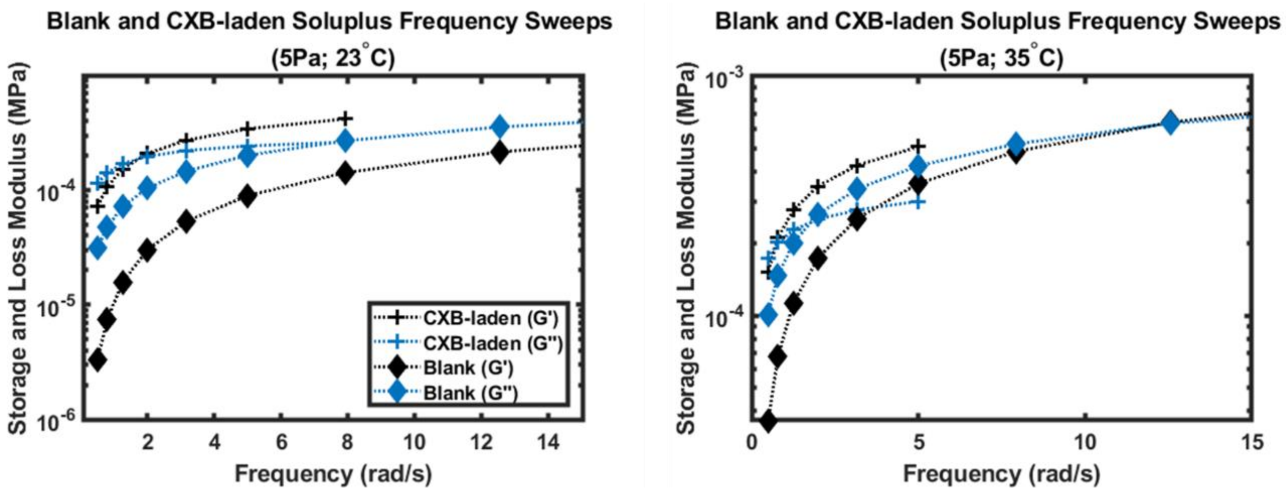

Fig. S5. Frequency sweeps at 5 Pa of blank and CXB-laden Soluplus samples. The frequency sweep on the left was done at 23°C and that on the right at 35°C.

Alginate Degradation in SBF

We measured alginate degradation in SBF by preparing 4 groups of samples. The first two groups were slabs (20 mm diameter, ~2 mm height) of 2.6% alginate (n=3) with or without addition of 0.1% LPEI (n=2; solution prepared as described in the main manuscript). These samples were crosslinked by submersion in 60 mL of 3 w/v% CaCl2 at 70°C for 24 hours. We chose this crosslinking time to ensure that crosslinking was complete and uniform. Following crosslinking, slabs from these two groups were placed into 6 mL of SBF at 37°C for 1 week.

Groups 3 and 4 were slabs (20 mm diameter, ~2mm height) of 2.6% alginate formulated as composites with CXB-laden Soluplus (as described in the main manuscript; n=3), with or without addition of 0.1% LPEI (n=2). These were crosslinked by submersion in 60 mL of 3 w/v% CaCl2 at 70°C for 30 minutes. Preliminary experiments conducted while designing the composite devices indicated no further stiffening past 30 minutes of crosslinking; we therefore considered it unnecessary to crosslink for longer. As we are primarily interested in characterizing the alginate, crosslinked slabs from these two groups were placed into 50 mL of DI water at 8°C for 3 days to allow for removal of Soluplus; previous work has shown ionically crosslinked alginate experiences essentially no degradation in Millipore water[4]. After these three days, they were then placed into 6 mL of SBF at 37°C for up to 2 weeks.

The complex moduli of the 4 groups described above were then measured using an Ares G2 rheometer for up to 2 weeks. Rheometry measurements were conducted at 37°C with a strain% of 0.5% and angular frequency of 6 rad/s (similar to the 0.5% strain and 1 Hz used previously[5]). The results

reported are the mean complex modulus calculated from 6 measurements taken over 60 seconds. None of the 4 groups tested demonstrated significant swelling over the testing period; slab heights varied by at most 100 microns (~5% of the initial 2 mm) during the testing period.

Figure S6A below shows the variation of complex modulus of the first two groups (not composited with Soluplus) over 1 week. As shown, these both start with a complex modulus of ~ 145 kPa. However, while the complex modulus of the pure alginate slabs falls to ~110 kPa after a week, that of the LPEI-reinforced alginate remains mostly unchanged/ increases slightly. This result indicates that addition of LPEI reduces alginate gel degradation (as has been previously shown[6]).

Figure S6B shows variation of the complex modulus of groups 3 and 4 (composited with Soluplus). The first three days (shaded in grey) represent the time spent in DI water to remove Soluplus. We expected that incubation in DI water would result in minimal degradation (due to the lack of sodium ions to exchange with crosslinking calcium ions[7]). However, we found that the alginate-Soluplus composites weakened significantly while in DI water (from an initial ~15 kPa to less than 10 kPa); we hypothesize that this was due to loss of structural support provided by Soluplus domains. Contrastingly, alginate-LPEI-Soluplus composites started with significantly higher modulus (over 40 kPa) and experienced very little softening during the DI water stage. Transition to SBF allowed continued softening of the alginate-Soluplus composites (to below 4 kPa after only 2 days; shown by the horizontal, dashed black line). The same transition caused a precipitous drop in the modulus of alginate-LPEI-Soluplus composites. However, these remained at ~10 kPa on day 2 and only fell by approximately 2 kPa to ~8kPa by day 14. We found that slabs with complex modulus below 4 kPa experienced permanent deformation even with gentle handling, and therefore chose to stop characterizing the alginate-Soluplus slabs at day 5.

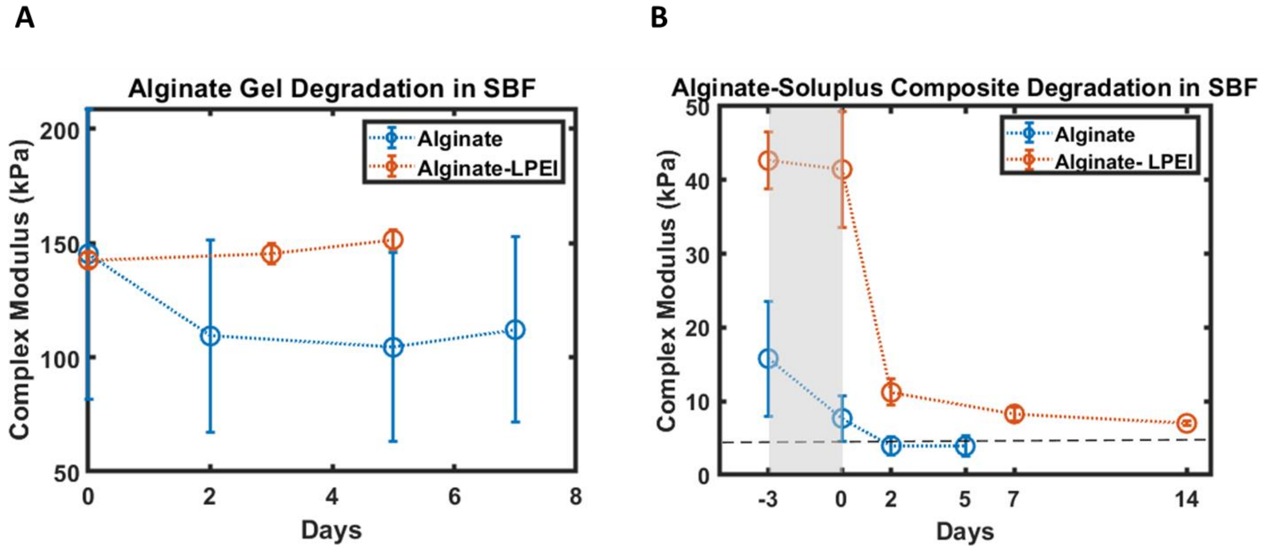

Fig. S6. A.) Variation in complex modulus of Alginate and Alginate-LPEI slabs over time. B.) Variation in complex moduli of Alginate and Alginate-LPEI slabs composited with CXB-laden Soluplus over time. The grey region represents time spent in DI water (to allow Soluplus release without degradation related to sodium ion exchange), and the horizontal, dashed black line represents 4 kPa complex modulus.

Cooling-Triggered Release of Additional Drugs

To further test our hypothesis that hydrophobic drugs with high pKa (~ 9 or greater) are the drugs (specifically, weak acid drugs) best suited for cooling-triggered release using our composite devices, we characterized cooling-triggered release of Indapamide (INP) and Estradiol (EST). These drugs are both hydrophobic weak acids; however, the pKa of Indapamide is 8.8, while that of Estradiol is 10.7. Note that for the purposes of this study, we limited the drugs tested to weak acids.

To briefly characterize cooling-triggered release of INP and EST, we prepared INP-laden and EST-laden Soluplus using the same procedure and water/polymer/drug ratios described for the CXB-laden Soluplus formulations. Next, we prepared composite devices as described in the main manuscript and washed them in SBF (also as described in the main manuscript). Finally, we followed the procedure outlined in the "*In vitro* cooling-triggered release" section of the Experimental Section to characterize one cooling-triggered release cycle of INP and EST. This involved maintaining the composite devices at 35°C for 2 days, then actively cooling them to 29°C for 40 minutes (all in 10 ml wells of SBF). The cooling-triggered increase in drug release was then observed out to 100 minutes after the initiation of the cooling trigger using a plate reader (similar procedure to that used for CXB measurement in the main manuscript, except Estradiol is measured at 280 nm, instead of 260 nm).

As shown in Fig. S7A, Indapamide experienced a relatively high leakage rate of ~12%/day while maintained at 35°C (OFF state). Additionally, upon cooling (ON state), the rate of Indapamide release only increased by ~6x. In contrast, Fig. S7B shows Estradiol experienced a leakage rate that averaged under 5%/day at 35°C (OFF state). Also, upon cooling, Estradiol's release rate increased by approximately ~20x (ON state); as stated in the main manuscript, that of CXB increased by ~30x.

These results strengthen our hypothesis as EST (pKa= 10.7) demonstrated good cooling-triggered release characteristics (low leakage in the OFF state and high ON/OFF ratio), while INP (pKa=8.8) demonstrated less robust cooling-triggered release characteristics. These data are also in agreement with the favorable release characteristics of both Curcumin (pKa values of 7.4, 9.6, and 10.9) and CXB (~11) and the less favorable cooling-triggered release characteristics of Indomethacin (pKa=4.5).

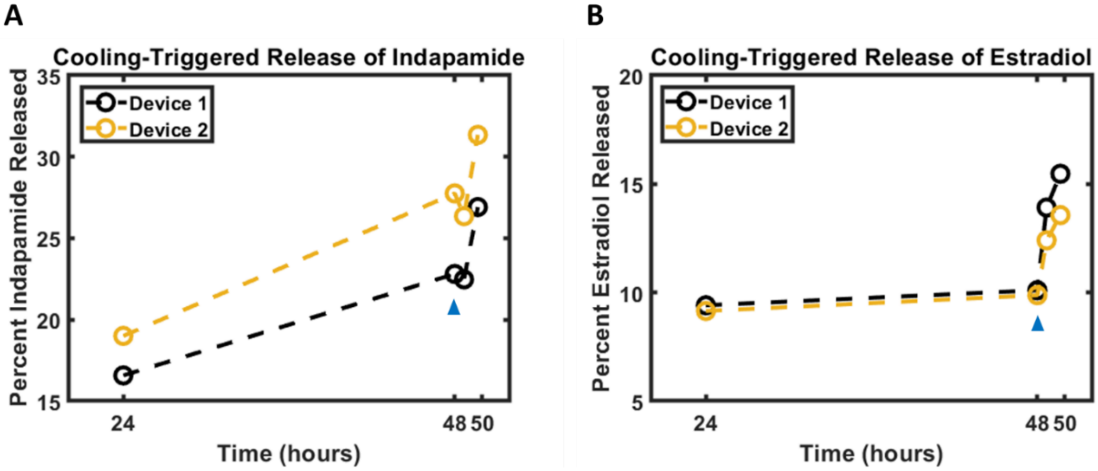

Fig. S7. Cooling-triggered release of A) INP and B) EST from composite devices in 10 ml wells of SBF. Blue triangles represent application of cooling as shown in Fig. 4C of the main manuscript. Measurements quantifying INP and EST are taken 40 minutes and 100 minutes after initiation of cooling.

Works Cited

(1) Mucha, I.; Karolewicz, B.; Górniak, A. Stability Studies of Amorphous Ibrutinib Prepared Using the Quench-Cooling Method and Its Dispersions with Soluplus®. *Polymers* **2024**, *16* (14), 1961. https://doi.org/10.3390/polym16141961.

(2) Cespi, M.; Casettari, L.; Palmieri, G. F.; Perinelli, D. R.; Bonacucina, G. Rheological Characterization of Polyvinyl Caprolactam–Polyvinyl Acetate–Polyethylene Glycol Graft Copolymer (Soluplus®) Water Dispersions. *Colloid Polym Sci* **2014**, *292* (1), 235–241. https://doi.org/10.1007/s00396-013-3077-8.

(3) Salah, I.; Shamat, M. A.; Cook, M. T. Soluplus Solutions as Thermothickening Materials for Topical Drug Delivery. *J of Applied Polymer Sci* **2019**, *136* (1), 46915. https://doi.org/10.1002/app.46915.

(4) Hunt, N. C.; Smith, A. M.; Gbureck, U.; Shelton, R. M.; Grover, L. M. Encapsulation of Fibroblasts Causes Accelerated Alginate Hydrogel Degradation. *Acta Biomaterialia* **2010**, *6* (9), 3649–3656. https://doi.org/10.1016/j.actbio.2010.03.026.

(5) Shahriari, D.; Koffler, J.; Lynam, D. A.; Tuszynski, M. H.; Sakamoto, J. S. Characterizing the Degradation of Alginate Hydrogel for Use in Multilumen Scaffolds for Spinal Cord Repair. *J Biomedical Materials Res* **2016**, *104* (3), 611–619. https://doi.org/10.1002/jbm.a.35600.

(6) Kong, H. J.; Mooney, D. J. The Effects of Poly(Ethyleneimine) (PEI) Molecular Weight on Reinforcement of Alginate Hydrogels. *Cell Transplant* **2003**, *12* (7), 779–785. https://doi.org/10.3727/000000003108747253.

(7) Zhang, X.; Huang, C.; Jin, X. Influence of K^+ and $N a^+$ Ions on the Degradation of Wet-spun Alginate Fibers for Tissue Engineering. *J of Applied Polymer Sci* **2017**, *134* (2), app.44396. https://doi.org/10.1002/app.44396.
